# Supplementary material for: Identifying candidate de novo genes expressed in the somatic female reproductive tract of Drosophila melanogaster
Source: bioRxiv. 2023 May 3:2023.05.03.539262. Preprint. [Version 1] doi: 10.1101/2023.05.03.539262 (PMC10187257; doi:10.1101/2023.05.03.539262)
Supplement: Supplement 1 [file NIHPP2023.05.03.539262v1-supplement-1.pdf]

bioRxiv preprint doi: https://doi.org/10.1101/2023.05.03.539262; this version posted May 3, 2023. The copyright holder for this preprint (which was not certified by peer review) is the author/funder, who has granted bioRxiv a license to display the preprint in perpetuity. It is made available under aCC-BY 4.0 International license.

SUPPLEMENTAL FIGURE 1 - Candidate de novo Gene Identification Pipeline Flowchart

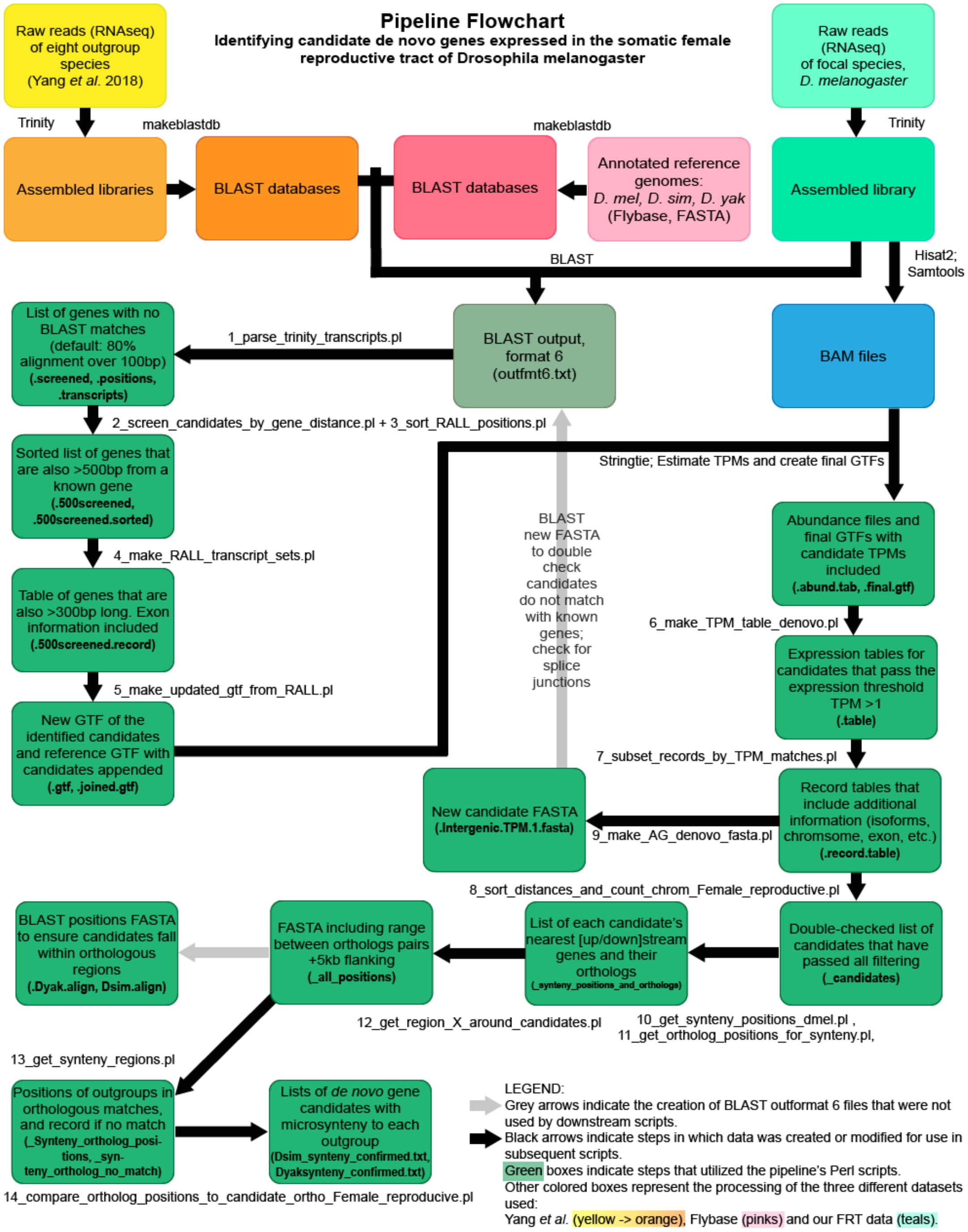

bioRxiv preprint doi: https://doi.org/10.1101/2023.05.03.539262; this version posted May 3, 2023. The copyright holder for this preprint (which was not certified by peer review) is the author/funder, who has granted bioRxiv a license to display the preprint in perpetuity. It is made available under aCC-BY 4.0 International license.

Supplemental Table 1 - Candidate TPMs in All FRT libraries

ID	RAL304 PV	RAL304 SR	RAL304 ST	RAL307xRAL304 F1 PV	RAL307xRAL304 F1 SR	RAL307cxRAL304 F1 ST	RAL307 PV	RAL307 SR	RAL307 ST	RAL360 SR	RAL360 ST	RAL399 ST	Chromosome Arm	# libraries TPM > 1	Average	Max	# libraries TPM > 0	TPM > 1 McDonough et al.	TPM > 1 FlyAtlas2	Potentially Coding
TRINITY DN18465 c0 g4	0.352788	1.415222	1.082299	0.538625	2.051786	0.642445	0.865103	1.955518	1.935462	0.507293	0.470648	0.402658	3R	5	1.018321	2.051786	12	YES	YES	-
TRINITY DN90 c0 g3	0.632434	0.080265	1.148475	1.404003	0.335493	1.04602	1.588585	0.602142	1.408332	0	0	0.177002	3R	5	0.701896	1.588585	10	-	-	CPC2
TRINITY DN52147 c0 g1	0.338275	1.186794	0	0	1.237453	0	0.168025	1.0116	0	1.063803	0	0	3L	4	0.417163	1.237453	6	YES	-	-
TRINITY DN2173 c0 g1	0	0.098262	0.861725	0.05266	0.040988	1.119179	0.048312	0.313738	6.996818	0	1.789367	0.731216	2L	3	1.004355	6.996818	10	YES	YES	-
TRINITY DN7862 c0 g3	0	3.287674	0	0	0	0	0	2.942715	0	1.182298	0	0	3R	3	0.617724	3.287674	3	-	-	CPAT
TRINITY DN4410 c0 g1	0.267146	0.149976	0.031198	0.613336	0.906185	0.320405	0	0.617751	0.088178	1.06928	1.22474	0.197922	2R	2	0.457176	1.22474	11	-	-	-
TRINITY DN72444 c0 g1	0.186571	0.133382	1.682349	0	0	0.820668	0.1202	0	0.915871	0.08394	1.516436	0.097806	3R	2	0.463102	1.682349	9	-	-	-
TRINITY DN16805 c0 g2	0	0	0.977034	0	0.327327	1.183629	0	0.065979	1.807839	0.053583	0.380538	0.130229	3L	2	0.410513	1.807839	8	YES	-	CPC2
TRINITY DN6533 c0 g1 *	0	1.520613	0	0	1.808348	0.236472	0	0.81718	0.36486	0.769066	0.087223	0.462391	2R	2	0.505513	1.808348	8	-	-	-
TRINITY DN4173 c1 g1	0	0.619289	0	0	1.495713	0.035867	0	1.124306	0	0.199292	0.1064	0	3R	2	0.298406	1.495713	6	-	-	-
TRINITY DN22183 c0 g1	0.14172	0.564105	0	0	1.046748	0	0	0.356866	0	1.648367	0	0	2R	2	0.313151	1.648367	5	-	-	-
TRINITY DN4094 c0 g1 **	0	1.07015	0	0	0.261327	0	0	1.089505	0	0.882769	0	0	2R	2	0.275313	1.089505	4	YES	-	-
TRINITY DN47998 c0 g1	0	0.812252	0	0	1.990564	0	0	0.326089	0	1.373493	0	0	3R	2	0.3752	1.990564	4	-	-	CPC2
TRINITY DN5611 c0 g1	0	0	0	0	0	0	0	0	1.414677	0	0.208555	2.468398	2R	2	0.340969	2.468398	3	YES	-	-
TRINITY DN7278 c0 g1	0	0.186198	0.248867	0.078581	0.077661	0.057982	0.078886	0.14183	0.234701	0	0.755092	1.069868	2R	1	0.244139	1.069868	10	-	YES	-
TRINITY DN58663 c0 g1 **	0.295466	0.46459	0	0.683942	0	0.471121	0.157672	1.063349	0	0.628004	0.086608	0	2R	1	0.320896	1.063349	8	YES	YES	-
TRINITY DN15918 c0 g3	0	0.054186	0.133204	0.101	0.099671	0.227033	0.119047	0	1.273411	0	0	0	X	1	0.167296	1.273411	7	-	-	-
TRINITY DN22384 c0 g1	0	0.089618	0.299821	0	0.219634	0.209227	0	0.028416	1.450873	0	0	0.011586	3L	1	0.192431	1.450873	7	-	-	-
TRINITY DN27150 c0 g4	0	0.108176	0.05758	0.399018	1.250246	0	0	0.237557	0.078213	0.050616	0	0	2L	1	0.181784	1.250246	7	-	-	-
TRINITY DN40913 c0 g1	0	0.263927	0.424273	0	0	0.643634	0.093435	0	0.383588	0	0.346142	1.229093	X	1	0.282008	1.229093	7	-	YES	-
TRINITY DN13179 c1 g1	0	0	0.085773	0	0.369057	0.17362	0	0	1.402391	0	0.089544	0.04869	2R	1	0.180756	1.402391	6	-	-	-
TRINITY DN5533 c0 g1	0	0	0.726944	0	0.110374	0.772581	0	0.200553	1.875943	0	0.129795	0	3L	1	0.318016	1.875943	6	-	-	-
TRINITY DN7830 c0 g1	0	0.052247	0	0.090456	0.25379	3.186478	0	0	0.119708	0	0	0	3L	1	0.308557	3.186478	5	-	YES	-
TRINITY DN24046 c0 g1	0.156638	0	0	0	0.223102	0	1.212832	0	0.103387	0	0	0	X	1	0.14133	1.212832	4	-	YES	-
TRINITY DN65427 c0 g1	0	0.268755	0	0	0.115866	0	0.064494	0	1.094541	0	0	0	3L	1	0.128638	1.094541	4	-	YES	-
TRINITY DN2143 c0 g2	0	0	0.225077	0	0	0.769195	0	0	1.625869	0	0	0.149369	X	1	0.230793	1.625869	4	-	YES	-
TRINITY DN65366 c0 g1 **	0	0	0	0	0.716048	0.135635	0	0	0	1.20102	0.383488	0	3L	1	0.203016	1.20102	4	-	-	-
TRINITY DN3265 c1 g1	0	0.513421	0	0	0.482888	0	0	1.264008	0	0.165274	0	0	3L	1	0.202133	1.264008	4	YES		-
TRINITY DN10735 c0 g1	0	1.146609	0	0	0.785431	0	0	0.48015	0	0.579941	0	0	2L	1	0.249344	1.146609	4	-	-	-
TRINITY DN11963 c0 g1	0	0	0	0	0	0.110392	0	0	0.169814	0	0	1.590221	2R	1	0.155869	1.590221	3	-	YES	-
TRINITY DN43648 c0 g1 *	0	0.636006	0	0	0.309986	0	0	0	0	1.401412	0	0	2R	1	0.195617	1.401412	3	-	-	-
TRINITY DN41677 c0 g1	0	0	0	1.014417	0	0	0.166003	0	0	0	0	0	3L	1	0.098368	1.014417	2	-	-	-
TRINITY DN66768 c0 g1	0	0	0	0.116518	0	1.430826	0	0	0	0	0	0	3L	1	0.128945	1.430826	2	-	YES	-
TRINITY DN55991 c0 g1	0	0	0	0	0	0	0	1.477049	0.109276	0	0	0	3R	1	0.132194	1.477049	2	-	-	-
TRINITY DN12518 c0 g2 *	0	0	0	0	1.026396	0	0	0	0	0.025473	0	0	3R	1	0.087656	1.026396	2	-	-	-
Max TPM per Library	0.632434	3.287674	1.682349	1.404003	2.051786	3.186478	1.588585	2.942715	6.996818	1.648367	1.789367	2.468398								

bioRxiv preprint doi: https://doi.org/10.1101/2023.05.03.539262; this version posted May 3, 2023. The copyright holder for this preprint (which was not certified by peer review) is the author/funder, who has granted bioRxiv a license to display the preprint in perpetuity. It is made available under a CC-BY 4.0 International license.

Supplemental Table 2 - TPMs of Accessory Gland Candidates with TPM ≥ 1 in All FRT Libraries

ID	RAL304 PV	RAL304 SR	RAL304 ST	RAL307xRAL304 F1 PV	RAL307xRAL304 F1 SR	RAL307cxRAL304 F1 ST	RAL307 PV	RAL307 SR	RAL307 ST	RAL360 SR	RAL360 ST	RAL399 ST
AG TRINITY DN1722 c1 g1	0.036302	0.095026	0.147355	0.222508	1.027393	0.26371	0.143937	0.166955	0.267048	0.266105	0	0
AG TRINITY DN6560 c0 g1	1.784064	2.312296	1.246803	0.941093	0.369655	0.304119	0	0	0	0	0	0
AG TRINITY DN43 c0 g1	0.201254	0.15081	0	0.57037	0.586483	1.447705	0	0.729053	0.359431	0.542643	0.123123	0.234664
AG TRINITY DN4095 c0 g2	0.750161	0.166792	0.42265	1.085351	0.343641	0.067001	0.922112	0	0.473483	0	0	0
AG TRINITY DN1423 c1 g1	0.146043	0	0	0.080036	0.67514	0.696276	0	0	0	2.125597	2.55081	0
AG TRINITY DN20247 c0 g2	0	0	0	0.109874	1.014351	0	0	0	0	0	0	0
AG TRINITY DN33386 c0 g1	0.481145	0.224157	0	1.297435	0.048004	0	0.756971	0.155309	0	0.210719	0	0
AG TRINITY DN31409 c0 g1	0.993161	1.098893	0	1.986941	2.303801	1.681559	1.208935	0.272492	2.312068	0.852267	0.136709	0
AG TRINITY DN6469 c0 g1	0.183608	0	0.403339	0.090745	0.281952	1.130011	0	0.282331	1.857159	0.065906	0.546285	1.292911
AG TRINITY DN5341 c0 g1	0.036992	0.125181	0.059539	0.140399	1.026885	0.199735	0.184515	0.791284	0.368757	0.037905	0	0.166803
AG TRINITY DN29646 c0 g1	0.517876	0.683924	0.877377	0.309203	0.571302	1.43811	0	0.157486	0.141442	0.310536	1.091643	0
AG TRINITY DN42840 c0 g1	0.489313	3.558292	1.022684	1.592972	3.379144	1.449994	0.685015	3.071053	1.68804	2.83442	1.663259	1.37938

bioRxiv preprint doi: https://doi.org/10.1101/2023.05.03.539262; this version posted May 3, 2023. The copyright holder for this preprint (which was not certified by peer review) is the author/funder, who has granted bioRxiv a license to display the preprint in perpetuity. It is made available under aCC-BY 4.0 International license.

Supplemental Table 3 - Raw Read Counts for All FRT Libraries

Raw Read Files (Left and Right)	Number of Reads
304_PV_S195_L003_R1_001.fastq.gz	20513346
304_PV_S195_L003_R2_001.fastq.gz	20513346
307_PV_S198_L003_R1_001.fastq.gz	16183938
307_PV_S198_L003_R2_001.fastq.gz	16183938
304_SR_S194_L003_R1_001.fastq.gz	19797634
304_SR_S194_L003_R2_001.fastq.gz	19797634
307_SR_S197_L003_R1_001.fastq.gz	14127659
307_SR_S197_L003_R2_001.fastq.gz	14127659
304_ST_S193_L003_R1_001.fastq.gz	18778304
304_ST_S193_L003_R2_001.fastq.gz	18778304
307_ST_S196_L003_R1_001.fastq.gz	21357080
307_ST_S196_L003_R2_001.fastq.gz	21357080
360_ST_S199_L003_R1_001.fastq.gz	18061012
360_ST_S199_L003_R2_001.fastq.gz	18061012
360_SR_S200_L003_R1_001.fastq.gz	15488884
360_SR_S200_L003_R2_001.fastq.gz	15488884
399_ST_S201_L003_R1_001.fastq.gz	17549951
399_ST_S201_L003_R2_001.fastq.gz	17549951
307304_PV_S210_L003_R1_001.fastq.gz	18656859
307304_PV_S210_L003_R2_001.fastq.gz	18656859
307304_SR_S209_L003_R1_001.fastq.gz	19029027
307304_SR_S209_L003_R2_001.fastq.gz	19029027
307304_ST_S208_L003_R1_001.fastq.gz	20341929
307304_ST_S208_L003_R2_001.fastq.gz	20341929
Tai18_PV_S207_L003_R1_001.fastq.gz	19049430
Tai18_PV_S207_L003_R2_001.fastq.gz	19049430
Tai18_SR_S206_L003_R1_001.fastq.gz	19596835
Tai18_SR_S207_L003_R2_001.fastq.gz	19596835
Tai18_ST_S205_L003_R1_001.fastq.gz	21481121
Tai18_ST_S205_L003_R2_001.fastq.gz	21481121
LARA10_PV_S204_L003_R1_001.fastq.gz	18161209
LARA10_PV_S204_L003_R2_001.fastq.gz	18161209
LARA10_SR_S203_L003_R1_001.fastq.gz	20153219
LARA10_SR_S203_L003_R2_001.fastq.gz	20153219
LARA10_ST_S202_L003_R1_001.fastq.gz	23805531
LARA10_ST_S202_L003_R2_001.fastq.gz	23805531

bioRxiv preprint doi: https://doi.org/10.1101/2023.05.03.539262; this version posted May 3, 2023. The copyright holder for this preprint (which was not certified by peer review) is the author/funder, who has granted bioRxiv a license to display the preprint in perpetuity. It is made available under aCC-BY 4.0 International license.

Supplemental Table 4 - Expressed Candidate TPMs in McDonough *et al.* Libraries

Gene ID	Chromosome	Strand	Start	End	Coverage	FPKM	TPM	Transcript
TRINITY_DN4094_c0_g1		-	20174519	20179171	5.186629	3.200418	2.369642	Seminal.receptacle.Unmated
TRINITY_DN4094_c0_g1		.	20178453	20179171	0	0	0	Seminal.receptacle.Unmated
TRINITY_DN4094_c0_g1		-	20174519	20179171	5.116992	2.596504	2.248902	Seminal.receptacle.Unmated
TRINITY_DN4094_c0_g1		.	20178453	20179171	0	0	0	Seminal.receptacle.Unmated
TRINITY_DN4094_c0_g1		-	20174519	20179171	4.309192	3.004301	2.340003	Seminal.receptacle.Unmated
TRINITY_DN4094_c0_g1		.	20178453	20179171	0	0	0	Seminal.receptacle.Unmated
TRINITY_DN52147_c0_g1		.	20572588	20572996	1.833741	2.523906	2.080056	Seminal.receptacle.24hr.Postmating
TRINITY_DN52147_c0_g1		.	20572588	20572996	4.026895	3.204057	2.601968	Seminal.receptacle.6hr.Postmating
TRINITY_DN52147_c0_g1		.	20572588	20572996	3.877751	2.877638	2.997787	Seminal.receptacle.6hr.Postmating
TRINITY_DN52147_c0_g1		.	20572588	20572996	5.408313	4.488916	3.323668	Seminal.receptacle.Unmated
TRINITY_DN52147_c0_g1		.	20572588	20572996	6.202934	4.963731	4.028016	Seminal.receptacle.6hr.Postmating
TRINITY_DN52147_c0_g1		.	20572588	20572996	5.466993	4.10235	3.553155	Seminal.receptacle.Unmated
TRINITY_DN52147_c0_g1		.	20572588	20572996	3.298289	2.463062	2.102138	Seminal.receptacle.6hr.Postmating
TRINITY_DN52147_c0_g1		.	20572588	20572996	5.041565	4.053394	3.157126	Seminal.receptacle.Unmated
TRINITY_DN52147_c0_g1		.	20572588	20572996	5.442543	4.066907	3.493271	Seminal.receptacle.24hr.Postmating
TRINITY_DN52147_c0_g1		.	20572588	20572996	2.682152	4.268307	3.423573	Seminal.receptacle.24hr.Postmating
TRINITY_DN2173_c0_g1		.	3492352	3493257	4.780353	3.81143	3.631062	Spermatheca.6hr.Postmating
TRINITY_DN2173_c0_g1		.	3492352	3493257	5.467991	4.685169	3.985463	Spermatheca.24hr.Postmating
TRINITY_DN2173_c0_g1		.	3492352	3493257	5.898455	5.039412	4.018377	Spermatheca.24hr.Postmating
TRINITY_DN2173_c0_g1		.	3492352	3493257	3.389625	2.902888	2.438463	Spermatheca.Unmated
TRINITY_DN2173_c0_g1		.	3492352	3493257	5.707506	4.549517	4.306667	Spermatheca.6hr.Postmating
TRINITY_DN2173_c0_g1		.	3492352	3493257	2.780353	2.384407	1.996818	Spermatheca.Unmated
TRINITY_DN2173_c0_g1		.	3492352	3493257	3.959161	3.155354	2.787655	Spermatheca.Unmated
TRINITY_DN2173_c0_g1		.	3492352	3493257	7.353201	5.893949	5.443974	Spermatheca.24hr.Postmating
TRINITY_DN2173_c0_g1		.	3492352	3493257	5.449227	4.339083	4.100268	Spermatheca.24hr.Postmating
TRINITY_DN2173_c0_g1		.	3492352	3493257	5.481236	4.682996	3.803852	Spermatheca.6hr.Postmating
TRINITY_DN2173_c0_g1		.	3492352	3493257	4.753863	3.783912	3.318386	Spermatheca.Unmated
TRINITY_DN2173_c0_g1		.	3492352	3493257	4.77925	4.093534	3.295012	Spermatheca.6hr.Postmating
TRINITY_DN16805_c0_g2		.	10619351	10620474	3.919039	3.119515	2.97189	Spermatheca.6hr.Postmating
TRINITY_DN16805_c0_g2		.	10619351	10620474	2.688612	2.371514	2.017341	Spermatheca.24hr.Postmating
TRINITY_DN16805_c0_g2		.	10619351	10620474	2.587189	2.332805	1.860156	Spermatheca.24hr.Postmating
TRINITY_DN16805_c0_g2		.	10619351	10620474	1.678826	1.483578	1.246225	Spermatheca.Unmated
TRINITY_DN16805_c0_g2		.	10619351	10620474	3.827402	3.209523	3.038202	Spermatheca.6hr.Postmating
TRINITY_DN16805_c0_g2		.	10619351	10620474	2.663701	2.510848	2.102705	Spermatheca.Unmated
TRINITY_DN16805_c0_g2		.	10619351	10620474	2.815836	2.19365	1.93802	Spermatheca.Unmated
TRINITY_DN16805_c0_g2		.	10619351	10620474	3.983986	3.200396	2.956061	Spermatheca.24hr.Postmating
TRINITY_DN16805_c0_g2		.	10619351	10620474	3.161032	2.59456	2.451761	Spermatheca.24hr.Postmating
TRINITY_DN16805_c0_g2		.	10619351	10620474	2.635231	2.504455	2.034291	Spermatheca.6hr.Postmating
TRINITY_DN16805_c0_g2		.	10619351	10620474	3.605872	2.860623	2.508687	Spermatheca.Unmated
TRINITY_DN16805_c0_g2		.	10619351	10620474	1.717972	1.632151	1.313769	Spermatheca.6hr.Postmating
TRINITY_DN58663_c0_g1		.	5173105	5173768	1.641566	1.203312	1.443088	Bursa.Unmated
TRINITY_DN58663_c0_g1		.	5173105	5173768	1.274096	1.00032	1.007925	Bursa.24hr.Postmating
TRINITY_DN58663_c0_g1		.	5173105	5173768	3.305723	2.632492	2.239343	Spermatheca.24hr.Postmating
TRINITY_DN58663_c0_g1		.	5173105	5173768	1.878012	2.584839	2.130274	Seminal.receptacle.24hr.Postmating
TRINITY_DN58663_c0_g1		.	5173105	5173768	1.621988	1.184027	1.259097	Oviduct.Unmated
TRINITY_DN58663_c0_g1		.	5173105	5173768	2.121988	1.680127	1.339716	Spermatheca.24hr.Postmating
TRINITY_DN58663_c0_g1		.	5173105	5173768	0.960843	0.762211	0.640267	Spermatheca.Unmated
TRINITY_DN58663_c0_g1		.	5173105	5173768	1.743976	1.373567	1.244502	Oviduct.6hr.Postmating
TRINITY_DN58663_c0_g1		.	5173105	5173768	1.036145	0.824424	0.669503	Seminal.receptacle.6hr.Postmating
TRINITY_DN58663_c0_g1		.	5173105	5173768	3.058735	2.269855	2.364627	Seminal.receptacle.6hr.Postmating
TRINITY_DN58663_c0_g1		.	5173105	5173768	1.510542	1.107869	1.302605	Bursa.24hr.Postmating
TRINITY_DN58663_c0_g1		.	5173105	5173768	2.468374	1.946906	1.785768	Oviduct.6hr.Postmating
TRINITY_DN58663_c0_g1		.	5173105	5173768	1.950301	1.560676	1.266472	Seminal.receptacle.6hr.Postmating
TRINITY_DN58663_c0_g1		.	5173105	5173768	1.71988	1.350474	1.445085	Bursa.24hr.Postmating
TRINITY_DN58663_c0_g1		.	5173105	5173768	1.204819	0.880627	1.032499	Bursa.Unmated
TRINITY_DN58663_c0_g1		.	5173105	5173768	2.025602	1.609468	1.347847	Spermatheca.Unmated
TRINITY_DN58663_c0_g1		.	5173105	5173768	1.057229	0.789506	0.673816	Seminal.receptacle.6hr.Postmating
TRINITY_DN58663_c0_g1		.	5173105	5173768	1.417169	1.047023	0.925012	Spermatheca.Unmated
TRINITY_DN58663_c0_g1		.	5173105	5173768	1.560241	1.165881	1.001434	Seminal.receptacle.24hr.Postmating
TRINITY_DN58663_c0_g1		.	5173105	5173768	1.424699	1.061579	0.980532	Spermatheca.24hr.Postmating
TRINITY_DN58663_c0_g1		.	5173105	5173768	1.198795	0.887597	0.838745	Spermatheca.24hr.Postmating
TRINITY_DN58663_c0_g1		.	5173105	5173768	1.927711	1.517427	1.383381	Oviduct.24hr.Postmating
TRINITY_DN58663_c0_g1		.	5173105	5173768	1.420181	1.115574	1.196117	Bursa.Unmated

bioRxiv preprint doi: https://doi.org/10.1101/2023.05.03.539262; this version posted May 3, 2023. The copyright holder for this preprint (which was not certified by peer review) is the author/funder, who has granted bioRxiv a license to display the preprint in perpetuity. It is made available under aCC-BY 4.0 International license.

TRINITY_DN58663_c0_g1	.	5173105	5173768	2.179217	1.608764	1.410841	Spermatheca.Unmated
TRINITY_DN58663_c0_g1	.	5173105	5173768	1.057229	0.77538	0.847179	Bursa.24hr.Postmating
TRINITY_DN58663_c0_g1	.	5173105	5173768	1.558735	1.226776	1.123026	Oviduct.24hr.Postmating
TRINITY_DN58663_c0_g1	.	5173105	5173768	1.798193	1.443493	1.453229	Oviduct.Unmated
TRINITY_DN58663_c0_g1	.	5173105	5173768	1.802711	1.429041	1.504771	Oviduct.Unmated
TRINITY_DN58663_c0_g1	.	5173105	5173768	2.159639	1.704824	1.901236	Bursa.Unmated
TRINITY_DN58663_c0_g1	.	5173105	5173768	1.646084	2.619535	2.101107	Seminal.receptacle.24hr.Postmating
TRINITY_DN58663_c0_g1	.	5173105	5173768	1.86747	1.415018	1.880928	Oviduct.24hr.Postmating
TRINITY_DN3265_c1_g1	+	6722731	6723168	1.403646	1.931936	1.592189	Seminal.receptacle.24hr.Postmating
TRINITY_DN3265_c1_g1	+	6722731	6723168	0.515625	0.410264	0.33317	Seminal.receptacle.6hr.Postmating
TRINITY_DN3265_c1_g1	+	6722731	6723168	2.044271	1.517032	1.580372	Seminal.receptacle.6hr.Postmating
TRINITY_DN3265_c1_g1	+	6722731	6723168	1.804688	1.444153	1.171915	Seminal.receptacle.6hr.Postmating
TRINITY_DN3265_c1_g1	+	6722731	6723168	1.458333	1.089039	0.929457	Seminal.receptacle.6hr.Postmating
TRINITY_DN3265_c1_g1	+	6722731	6723168	2.210938	1.65211	1.41908	Seminal.receptacle.24hr.Postmating
TRINITY_DN3265_c1_g1	+	6722731	6723168	0	0	0	Seminal.receptacle.24hr.Postmating
TRINITY_DN5611_c0_g1	+	8273193	8274050	8.562737	2.940839	2.80167	Spermatheca.6hr.Postmating
TRINITY_DN5611_c0_g1	.	8273483	8274050	0	0	0	Spermatheca.6hr.Postmating
TRINITY_DN5611_c0_g1	+	8273193	8274050	7.74905	2.656945	2.515119	Spermatheca.6hr.Postmating
TRINITY_DN5611_c0_g1	.	8273483	8274050	0	0	0	Spermatheca.6hr.Postmating
TRINITY_DN5611_c0_g1	+	8273193	8274050	10.15589	3.753879	3.04916	Spermatheca.6hr.Postmating
TRINITY_DN5611_c0_g1	.	8273483	8274050	0	0	0	Spermatheca.6hr.Postmating
TRINITY_DN5611_c0_g1	+	8273193	8274050	0	0	0	Spermatheca.6hr.Postmating
TRINITY_DN5611_c0_g1	.	8273483	8274050	4.114437	3.918437	3.154071	Spermatheca.6hr.Postmating
TRINITY_DN18465_c0_g4	.	7091545	7091818	2.507299	1.994968	1.620085	Seminal.receptacle.6hr.Postmating
TRINITY_DN18465_c0_g4	.	7091545	7091818	1	0.735496	0.79176	Bursa.6hr.Postmating
TRINITY_DN18465_c0_g4	.	7091545	7091818	0.620438	0.46042	0.479644	Seminal.receptacle.6hr.Postmating
TRINITY_DN18465_c0_g4	.	7091545	7091818	2.839416	2.272166	1.843839	Seminal.receptacle.6hr.Postmating
TRINITY_DN18465_c0_g4	.	7091545	7091818	1.284672	1.008246	1.013419	Bursa.6hr.Postmating
TRINITY_DN18465_c0_g4	.	7091545	7091818	1.456204	1.087449	0.9281	Seminal.receptacle.6hr.Postmating
TRINITY_DN18465_c0_g4	.	7091545	7091818	1.635036	1.294636	1.299285	Bursa.6hr.Postmating
TRINITY_DN18465_c0_g4	.	7091545	7091818	1.324818	0.97144	1.154888	Bursa.6hr.Postmating
AG_TRINITY_DN4679_c0_g:	.	21856948	21857826	3.009153	2.20579	2.645322	Bursa.Unmated
AG_TRINITY_DN4679_c0_g:	.	21856948	21857826	1.154152	0.906149	0.913039	Bursa.24hr.Postmating
AG_TRINITY_DN4679_c0_g:	.	21856948	21857826	1.984073	1.448343	1.540172	Oviduct.Unmated
AG_TRINITY_DN4679_c0_g:	.	21856948	21857826	2.960294	2.331547	2.112468	Oviduct.6hr.Postmating
AG_TRINITY_DN4679_c0_g:	.	21856948	21857826	1.977247	1.573225	1.277593	Seminal.receptacle.6hr.Postmating
AG_TRINITY_DN4679_c0_g:	.	21856948	21857826	3.112628	2.289327	2.464454	Bursa.6hr.Postmating
AG_TRINITY_DN4679_c0_g:	.	21856948	21857826	1.474972	1.094561	1.140262	Seminal.receptacle.6hr.Postmating
AG_TRINITY_DN4679_c0_g:	.	21856948	21857826	1.899317	1.393006	1.637862	Bursa.24hr.Postmating
AG_TRINITY_DN4679_c0_g:	.	21856948	21857826	2.920363	2.303408	2.112763	Oviduct.6hr.Postmating
AG_TRINITY_DN4679_c0_g:	.	21856948	21857826	1.90273	1.579272	1.169319	Seminal.receptacle.Unmated
AG_TRINITY_DN4679_c0_g:	.	21856948	21857826	2.792378	2.234525	1.813294	Seminal.receptacle.6hr.Postmating
AG_TRINITY_DN4679_c0_g:	.	21856948	21857826	1.473265	1.156828	1.237873	Bursa.24hr.Postmating
AG_TRINITY_DN4679_c0_g:	.	21856948	21857826	1.990296	1.56204	1.570053	Bursa.6hr.Postmating
AG_TRINITY_DN4679_c0_g:	.	21856948	21857826	3.918657	2.864226	3.358188	Bursa.Unmated
AG_TRINITY_DN4679_c0_g:	.	21856948	21857826	1.415813	1.062405	0.920177	Seminal.receptacle.Unmated
AG_TRINITY_DN4679_c0_g:	.	21856948	21857826	1.807718	1.349949	1.152135	Seminal.receptacle.6hr.Postmating
AG_TRINITY_DN4679_c0_g:	.	21856948	21857826	1.653584	1.329474	1.035506	Seminal.receptacle.Unmated
AG_TRINITY_DN4679_c0_g:	.	21856948	21857826	1.792098	1.410678	1.286061	Oviduct.24hr.Postmating
AG_TRINITY_DN4679_c0_g:	.	21856948	21857826	3.159846	2.482108	2.661313	Bursa.Unmated
AG_TRINITY_DN4679_c0_g:	.	21856948	21857826	1.919209	1.519647	1.525104	Bursa.6hr.Postmating
AG_TRINITY_DN4679_c0_g:	.	21856948	21857826	1.960403	1.437775	1.57091	Bursa.24hr.Postmating
AG_TRINITY_DN4679_c0_g:	.	21856948	21857826	1.992647	1.568279	1.435647	Oviduct.24hr.Postmating
AG_TRINITY_DN4679_c0_g:	.	21856948	21857826	2.352674	1.888601	1.901339	Oviduct.Unmated
AG_TRINITY_DN4679_c0_g:	.	21856948	21857826	2.032423	1.611137	1.696518	Oviduct.Unmated
AG_TRINITY_DN4679_c0_g:	.	21856948	21857826	2.122867	1.55662	1.850574	Bursa.6hr.Postmating
AG_TRINITY_DN4679_c0_g:	.	21856948	21857826	2.998862	2.367309	2.640045	Bursa.Unmated
AG_TRINITY_DN4679_c0_g:	.	21856948	21857826	1.782139	1.350361	1.794982	Oviduct.24hr.Postmating
AG_TRINITY_DN42840_c0_	+	24811204	24811908	1.056738	0.774617	0.92897	Bursa.Unmated
AG_TRINITY_DN42840_c0_	+	24811204	24811908	1.324823	0.979194	0.932856	Spermatheca.6hr.Postmating
AG_TRINITY_DN42840_c0_	+	24811204	24811908	2.746099	2.186839	1.860246	Spermatheca.24hr.Postmating
AG_TRINITY_DN42840_c0_	+	24811204	24811908	0.988652	1.360751	1.121451	Seminal.receptacle.24hr.Postmating
AG_TRINITY_DN42840_c0_	+	24811204	24811908	4.584397	3.34654	3.558719	Oviduct.Unmated
AG_TRINITY_DN42840_c0_	+	24811204	24811908	3.056738	2.420234	1.92987	Spermatheca.24hr.Postmating

bioRxiv preprint doi: https://doi.org/10.1101/2023.05.03.539262; this version posted May 3, 2023. The copyright holder for this preprint (which was not certified by peer review) is the author/funder, who has granted bioRxiv a license to display the preprint in perpetuity. It is made available under aCC-BY 4.0 International license.

AG_TRINITY_DN42840_c0_	+	24811204	24811908	2.506383	1.988246	1.670152	Spermatheca.Unmated
AG_TRINITY_DN42840_c0_	+	24811204	24811908	1.638298	1.213164	1.148407	Spermatheca.6hr.Postmating
AG_TRINITY_DN42840_c0_	+	24811204	24811908	4.788652	3.771574	3.417185	Oviduct.6hr.Postmating
AG_TRINITY_DN42840_c0_	+	24811204	24811908	2.948936	2.346363	1.905448	Seminal.receptacle.6hr.Postmating
AG_TRINITY_DN42840_c0_	+	24811204	24811908	1.551773	1.141323	1.228632	Bursa.6hr.Postmating
AG_TRINITY_DN42840_c0_	+	24811204	24811908	2.392908	1.775752	1.849894	Seminal.receptacle.6hr.Postmating
AG_TRINITY_DN42840_c0_	+	24811204	24811908	5.720567	4.512043	4.138597	Oviduct.6hr.Postmating
AG_TRINITY_DN42840_c0_	+	24811204	24811908	3.873759	3.215232	2.38061	Seminal.receptacle.Unmated
AG_TRINITY_DN42840_c0_	+	24811204	24811908	3.17305	2.539148	2.060492	Seminal.receptacle.6hr.Postmating
AG_TRINITY_DN42840_c0_	+	24811204	24811908	1.541844	1.210082	1.21629	Bursa.6hr.Postmating
AG_TRINITY_DN42840_c0_	+	24811204	24811908	1.275177	0.932053	1.092794	Bursa.Unmated
AG_TRINITY_DN42840_c0_	+	24811204	24811908	2.258156	1.794247	1.502589	Spermatheca.Unmated
AG_TRINITY_DN42840_c0_	+	24811204	24811908	2.665248	1.999963	1.732221	Seminal.receptacle.Unmated
AG_TRINITY_DN42840_c0_	+	24811204	24811908	2.395745	1.789069	1.526909	Seminal.receptacle.6hr.Postmating
AG_TRINITY_DN42840_c0_	+	24811204	24811908	0.851064	0.628777	0.555505	Spermatheca.Unmated
AG_TRINITY_DN42840_c0_	+	24811204	24811908	3.168794	2.547695	1.98436	Seminal.receptacle.Unmated
AG_TRINITY_DN42840_c0_	+	24811204	24811908	2.689362	2.009609	1.726155	Seminal.receptacle.24hr.Postmating
AG_TRINITY_DN42840_c0_	+	24811204	24811908	2.001418	1.491306	1.377452	Spermatheca.24hr.Postmating
AG_TRINITY_DN42840_c0_	+	24811204	24811908	1.197163	0.886389	0.837604	Spermatheca.24hr.Postmating
AG_TRINITY_DN42840_c0_	+	24811204	24811908	3.880851	3.054871	2.78501	Oviduct.24hr.Postmating
AG_TRINITY_DN42840_c0_	+	24811204	24811908	1.828369	1.436212	1.539905	Bursa.Unmated
AG_TRINITY_DN42840_c0_	+	24811204	24811908	1.26383	1.000712	1.004305	Bursa.6hr.Postmating
AG_TRINITY_DN42840_c0_	+	24811204	24811908	1.964539	1.560851	1.26783	Spermatheca.6hr.Postmating
AG_TRINITY_DN42840_c0_	+	24811204	24811908	1.191489	0.879593	0.771379	Spermatheca.Unmated
AG_TRINITY_DN42840_c0_	+	24811204	24811908	4.236879	3.334564	3.052555	Oviduct.24hr.Postmating
AG_TRINITY_DN42840_c0_	+	24811204	24811908	4.388653	3.522975	3.546737	Oviduct.Unmated
AG_TRINITY_DN42840_c0_	+	24811204	24811908	4.649645	3.685855	3.881183	Oviduct.Unmated
AG_TRINITY_DN42840_c0_	+	24811204	24811908	0.703546	0.515884	0.613304	Bursa.6hr.Postmating
AG_TRINITY_DN42840_c0_	+	24811204	24811908	2.150355	1.708019	1.374837	Spermatheca.6hr.Postmating
AG_TRINITY_DN42840_c0_	+	24811204	24811908	1.695035	1.338065	1.492222	Bursa.Unmated
AG_TRINITY_DN42840_c0_	+	24811204	24811908	1.978723	3.148889	2.525697	Seminal.receptacle.24hr.Postmating
AG_TRINITY_DN42840_c0_	+	24811204	24811908	3.039716	2.303252	3.061622	Oviduct.24hr.Postmating

bioRxiv preprint doi: https://doi.org/10.1101/2023.05.03.539262; this version posted May 3, 2023. The copyright holder for this preprint (which was not certified by peer review) is the author/funder, who has granted bioRxiv a license to display the preprint in perpetuity. It is made available under aCC-BY 4.0 International license.

Supplemental Table 5 - Expressed Candidate TPMs in FlyAtlas2 Libraries

Gene ID	Chromosome	Strand	Start	End	Coverage	FPKM	TPM	Gene Name	Reference	Transcript
TRINITY_DN2173_c0_g1		.	3492352	3493257	26.274834	4.176114	8.065937	-	2L	Male_Eye_2
TRINITY_DN2173_c0_g1		.	3492352	3493257	1.571744	0.339742	0.819945	-	2L	Male_Eye_3
TRINITY_DN2173_c0_g1		.	3492352	3493257	8.974614	1.318929	2.272738	-	2L	Male_Eye_1
TRINITY_DN2173_c0_g1		.	3492352	3493257	1.192053	0.286605	0.749776	-	2L	Female_Eye_3
TRINITY_DN2173_c0_g1		.	3492352	3493257	6.131348	0.939579	1.51707	-	2L	Female_Eye_2
TRINITY_DN2173_c0_g1		.	3492352	3493257	4.263181	0.611167	0.935139	-	2L	Female_Eye_1
TRINITY_DN66768_c0_g1		.	2.2E+07	2.2E+07	7.974127	5.229924	6.556244	-	3L	Female_Anal_Pad_2
TRINITY_DN66768_c0_g1		.	2.2E+07	2.2E+07	9.467011	5.844386	7.488165	-	3L	Female_Anal_Pad_3
TRINITY_DN66768_c0_g1		.	2.2E+07	2.2E+07	5.037516	3.483253	3.871449	-	3L	Male_Anal_Pad_3
TRINITY_DN66768_c0_g1		.	2.2E+07	2.2E+07	7.043984	4.407676	5.657084	-	3L	Male_Anal_Pad_1
TRINITY_DN66768_c0_g1		.	2.2E+07	2.2E+07	6.494178	4.072696	4.772302	-	3L	Female_Anal_Pad_1
TRINITY_DN66768_c0_g1		.	2.2E+07	2.2E+07	8.397154	5.239401	6.621044	-	3L	Male_Anal_Pad_2
TRINITY_DN65427_c0_g1		.	9903975	9904615	3.767551	1.893619	3.195135	-	3L	Male_Brain_2
TRINITY_DN65427_c0_g1		.	9903975	9904615	2.99376	0.418265	0.704603	-	3L	Female_Thoracicoabdominal_Ganglion_1
TRINITY_DN65427_c0_g1		.	9903975	9904615	3.224649	1.482536	2.526667	-	3L	Male_Brain_3
TRINITY_DN65427_c0_g1		.	9903975	9904615	6.670827	0.98156	1.710683	-	3L	Female_Thoracicoabdominal_Ganglion_2
TRINITY_DN65427_c0_g1		.	9903975	9904615	2.338534	1.077464	1.461239	-	3L	Female_Brain_3
TRINITY_DN65427_c0_g1		.	9903975	9904615	1.085803	0.550404	0.892902	-	3L	Female_Brain_1
TRINITY_DN65427_c0_g1		.	9903975	9904615	1.096724	0.548696	0.951509	-	3L	Male_Brain_1
TRINITY_DN65427_c0_g1		.	9903975	9904615	3.279251	1.578201	2.616482	-	3L	Female_Brain_2
TRINITY_DN24046_c0_g1		.	9119350	9119683	1.766467	0.813888	1.103781	-	X	Female_Brain_3
TRINITY_DN24046_c0_g1		.	9119350	9119683	1.431138	0.725458	1.176885	-	X	Female_Brain_1
TRINITY_DN24046_c0_g1		.	9119350	9119683	1.017964	0.489914	0.812223	-	X	Female_Brain_2
TRINITY_DN7830_c0_g1		+	2.2E+07	2.2E+07	11.81948	6.779947	8.499356	-	3L	Female_Anal_Pad_2
TRINITY_DN7830_c0_g1		+	2.2E+07	2.2E+07	16.846922	9.087433	11.643346	-	3L	Female_Anal_Pad_3
TRINITY_DN7830_c0_g1		+	2.2E+07	2.2E+07	5.503688	3.386535	3.763952	-	3L	Male_Anal_Pad_3
TRINITY_DN7830_c0_g1		+	2.2E+07	2.2E+07	6.494936	3.631603	4.661024	-	3L	Male_Anal_Pad_1
TRINITY_DN7830_c0_g1		+	2.2E+07	2.2E+07	7.792578	4.340876	5.086549	-	3L	Female_Anal_Pad_1
TRINITY_DN7830_c0_g1		+	2.2E+07	2.2E+07	4.83967	2.65907	3.360273	-	3L	Male_Anal_Pad_2
TRINITY_DN2143_c0_g2		.	9500809	9501829	1.851126	0.548945	1.300799	-	X	Female_Virgin_Spermatheca_1
TRINITY_DN2143_c0_g2		.	9500809	9501829	2.567091	0.593933	1.390857	-	X	Female_Virgin_Spermatheca_3
TRINITY_DN2143_c0_g2		.	9500809	9501829	4.56905	0.810354	1.94117	-	X	Female_Mated_Spermatheca_3
TRINITY_DN2143_c0_g2		.	9500809	9501829	4.047992	1.159555	2.734515	-	X	Female_Virgin_Spermatheca_2
TRINITY_DN2143_c0_g2		.	9500809	9501829	2.938296	0.736011	1.767465	-	X	Female_Mated_Spermatheca_2
TRINITY_DN2143_c0_g2		.	9500809	9501829	2.955925	0.565022	1.347279	-	X	Female_Mated_Spermatheca_1
TRINITY_DN7278_c0_g1		.	2.3E+07	2.3E+07	6.295321	3.558365	2.964927	-	2R	Male_Accessory_Glands_2
TRINITY_DN7278_c0_g1		.	2.3E+07	2.3E+07	1.991228	1.053764	0.904118	-	2R	Male_Accessory_Glands_1
TRINITY_DN7278_c0_g1		.	2.3E+07	2.3E+07	4.197369	2.160995	2.003997	-	2R	Male_Accessory_Glands_3
TRINITY_DN11963_c0_g1		.	9033508	9034113	2.331683	0.907222	1.375922	-	2R	Male_Hindgut_2
TRINITY_DN11963_c0_g1		.	9033508	9034113	0.957096	0.574778	0.832837	-	2R	Male_Hindgut_1
TRINITY_DN11963_c0_g1		.	9033508	9034113	1.382838	0.80649	1.320086	-	2R	Male_Hindgut_3
TRINITY_DN40913_c0_g1		+	2E+07	2E+07	1.349398	0.678225	1.14438	-	X	Male_Brain_2
TRINITY_DN40913_c0_g1		+	2E+07	2E+07	11.655957	1.628482	2.743313	-	X	Female_Thoracicoabdominal_Ganglion_1
TRINITY_DN40913_c0_g1		+	2E+07	2E+07	14.659973	2.129849	3.583353	-	X	Male_Thoracicoabdominal_Ganglion_1
TRINITY_DN40913_c0_g1		+	2E+07	2E+07	1.290495	0.593307	1.011165	-	X	Male_Brain_3
TRINITY_DN40913_c0_g1		+	2E+07	2E+07	7.155288	1.052845	1.834919	-	X	Female_Thoracicoabdominal_Ganglion_2
TRINITY_DN40913_c0_g1		+	2E+07	2E+07	1.72423	0.862639	1.495928	-	X	Male_Brain_1
TRINITY_DN40913_c0_g1		+	2E+07	2E+07	10.64257	1.663993	2.188475	-	X	Male_Thoracicoabdominal_Ganglion_2
TRINITY_DN58663_c0_g1		.	5173105	5173768	5.295197	0.739805	1.246263	-	2R	Female_Thoracicoabdominal_Ganglion_1
TRINITY_DN58663_c0_g1		.	5173105	5173768	1.924699	1.822181	2.667561	-	2R	Male_Whole_2
TRINITY_DN58663_c0_g1		.	5173105	5173768	0.5	0.498801	0.817612	-	2R	Male_Midgut_1
TRINITY_DN58663_c0_g1		.	5173105	5173768	1.206325	1.127514	1.790125	-	2R	Male_Midgut_3
TRINITY_DN58663_c0_g1		.	5173105	5173768	6.646084	0.965565	1.624509	-	2R	Male_Thoracicoabdominal_Ganglion_1
TRINITY_DN58663_c0_g1		.	5173105	5173768	0.891566	0.736702	0.926913	-	2R	Male_Whole_3
TRINITY_DN58663_c0_g1		.	5173105	5173768	0.251506	0.25032	0.396691	-	2R	Male_Midgut_2
TRINITY_DN58663_c0_g1		.	5173105	5173768	6.037651	0.888393	1.54831	-	2R	Female_Thoracicoabdominal_Ganglion_2
TRINITY_DN58663_c0_g1		.	5173105	5173768	0.849398	0.523312	0.592393	-	2R	Male_Whole_1
TRINITY_DN58663_c0_g1		.	5173105	5173768	2.813253	0.439859	0.578501	-	2R	Male_Thoracicoabdominal_Ganglion_2
TRINITY_DN18465_c0_g4		.	7091545	7091818	0.405109	0.212227	0.316536	-	3R	FemaleCarcass_3-2
TRINITY_DN18465_c0_g4		.	7091545	7091818	0.606296	0.333792	0.525552	-	3R	FemaleCarcass_1-1
TRINITY_DN18465_c0_g4		.	7091545	7091818	10.79562	5.426017	9.155407	-	3R	Male_Brain_2
TRINITY_DN18465_c0_g4		.	7091545	7091818	7.463504	6.249868	10.94217	-	3R	Larval_CNS_1
TRINITY_DN18465_c0_g4		.	7091545	7091818	1.572993	1.951726	2.768055	-	3R	Female_Whole_1
TRINITY_DN18465_c0_g4		.	7091545	7091818	4.339416	3.205006	6.000449	-	3R	Larval_CNS_3
TRINITY_DN18465_c0_g4		.	7091545	7091818	2.310476	1.344332	1.904483	-	3R	FemaleCarcass_2-2
TRINITY_DN18465_c0_g4		.	7091545	7091818	21.233574	2.966594	4.997475	-	3R	Female_Thoracicoabdominal_Ganglion_1
TRINITY_DN18465_c0_g4		.	7091545	7091818	21.177084	3.182338	6.146514	-	3R	Male_Eye_2
TRINITY_DN18465_c0_g4		.	7091545	7091818	2.711679	1.200012	1.490906	-	3R	Female_Crop_2
TRINITY_DN18465_c0_g4		.	7091545	7091818	28.193428	4.096033	6.89135	-	3R	Male_Thoracicoabdominal_Ganglion_1
TRINITY_DN18465_c0_g4		.	7091545	7091818	2.817518	2.613943	2.677556	-	3R	Female_Head_3
TRINITY_DN18465_c0_g4		.	7091545	7091818	3.587591	0.742469	1.7919	-	3R	Male_Eye_3
TRINITY_DN18465_c0_g4		.	7091545	7091818	15.521898	7.136212	12.162151	-	3R	Male_Brain_3
TRINITY_DN18465_c0_g4		.	7091545	7091818	6.835766	7.12184	12.63049	-	3R	Larval_CNS_2
TRINITY_DN18465_c0_g4		.	7091545	7091818	26.967207	3.754663	6.469921	-	3R	Male_Eye_1

bioRxiv preprint doi: https://doi.org/10.1101/2023.05.03.539262; this version posted May 3, 2023. The copyright holder for this preprint (which was not certified by peer review) is the author/funder, who has granted bioRxiv a license to display the preprint in perpetuity. It is made available under aCC-BY 4.0 International license.

TRINITY_DN18465_c0_g4	.	7091545	7091818	1.740876	0.921416	1.37703	-	3R	FemaleCarcass_3-1
TRINITY_DN18465_c0_g4	.	7091545	7091818	4.481752	0.9283	1.238712	-	3R	Female_Crop_1
TRINITY_DN18465_c0_g4	.	7091545	7091818	0.890511	0.96056	0.938929	-	3R	Female_Head_2
TRINITY_DN18465_c0_g4	.	7091545	7091818	23.846716	3.508859	6.115308	-	3R	Female_Thoracicoabdominal_Ganglion_2
TRINITY_DN18465_c0_g4	.	7091545	7091818	11.260036	5.187988	7.035862	-	3R	Female_Brain_3
TRINITY_DN18465_c0_g4	.	7091545	7091818	0.277885	0.164176	0.232732	-	3R	FemaleCarcass_2-1
TRINITY_DN18465_c0_g4	.	7091545	7091818	2.806569	0.629887	1.647822	-	3R	Female_Eye_3
TRINITY_DN18465_c0_g4	.	7091545	7091818	2.810219	1.407858	2.111621	-	3R	Larval_Trachea_3
TRINITY_DN18465_c0_g4	.	7091545	7091818	3.788321	4.136505	4.788397	-	3R	Male_Head_3
TRINITY_DN18465_c0_g4	.	7091545	7091818	1.29562	1.164333	1.731779	-	3R	Larval_Trachea_1
TRINITY_DN18465_c0_g4	.	7091545	7091818	26.530737	3.863672	6.238387	-	3R	Female_Eye_2
TRINITY_DN18465_c0_g4	.	7091545	7091818	0.886861	0.675609	0.943846	-	3R	Female_Whole_3
TRINITY_DN18465_c0_g4	.	7091545	7091818	7.761405	3.934334	6.382532	-	3R	Female_Brain_1
TRINITY_DN18465_c0_g4	.	7091545	7091818	0.386861	0.366983	0.616603	-	3R	Female_Ovary_2
TRINITY_DN18465_c0_g4	.	7091545	7091818	1.051095	0.752403	0.87388	-	3R	Male_Head_1
TRINITY_DN18465_c0_g4	.	7091545	7091818	6.043368	0.938611	1.377324	-	3R	Male_Crop_2
TRINITY_DN18465_c0_g4	.	7091545	7091818	0	0	0	-	3R	Male_Brain_1
TRINITY_DN18465_c0_g4	.	7091545	7091818	2.076642	1.12938	1.776592	-	3R	FemaleCarcass_1-2
TRINITY_DN18465_c0_g4	.	7091545	7091818	0.689781	0.598155	0.835353	-	3R	Larval_Trachea_2
TRINITY_DN18465_c0_g4	.	7091545	7091818	25.645016	3.498549	5.353084	-	3R	Female_Eye_1
TRINITY_DN18465_c0_g4	.	7091545	7091818	0.671533	0.585219	0.764926	-	3R	Female_Whole_2
TRINITY_DN18465_c0_g4	.	7091545	7091818	9.478102	4.561514	7.562485	-	3R	Female_Brain_2
TRINITY_DN18465_c0_g4	.	7091545	7091818	1.284672	1.183173	1.938949	-	3R	Female_Ovary_1
TRINITY_DN18465_c0_g4	.	7091545	7091818	4.142336	3.859741	5.230494	-	3R	Female_Head_1
TRINITY_DN18465_c0_g4	.	7091545	7091818	1.328467	1.190214	1.817249	-	3R	Female_Ovary_3
TRINITY_DN18465_c0_g4	.	7091545	7091818	1.124088	0.929364	1.545611	-	3R	Male_Crop_1
TRINITY_DN18465_c0_g4	.	7091545	7091818	3.167883	2.338553	2.51005	-	3R	Male_Head_2
TRINITY_DN18465_c0_g4	.	7091545	7091818	18.020159	2.817499	3.705558	-	3R	Male_Thoracicoabdominal_Ganglion_2
						12.63049			

bioRxiv preprint doi: https://doi.org/10.1101/2023.05.03.539262; this version posted May 3, 2023. The copyright holder for this preprint (which was not certified by peer review) is the author/funder, who has granted bioRxiv a license to display the preprint in perpetuity. It is made available under aCC-BY 4.0 International license.

Supplemental Table 6 - List of All BLAST Databases Utilized

Flybase References		Yang et al. References	
dana-3prime-r1-06	Drosophila-ananassae-female-abdomen-without-digestive-or-reproductive-system	Drosophila-pseudoobscura-female-abdomen-without-digestive-or-reproductive-system	Drosophila-willistoni-female-gonad
dana-5prime-r1-06	Drosophila-ananassae-female-digestive-plus-excretory-system	Drosophila-pseudoobscura-female-digestive-plus-excretory-system	Drosophila-willistoni-female-digestive-plus-excretory-system
dana-CDS-r1-06	Drosophila-ananassae-female-gonad	Drosophila-pseudoobscura-female-gonad	Drosophila-willistoni-female-reproductive-system-without-gonad
dana-miRNA-r1-06	Drosophila-ananassae-female-head	Drosophila-pseudoobscura-female-head	Drosophila-willistoni-female-head
dana-intron-r1-06	Drosophila-ananassae-female-reproductive-system-without-gonad	Drosophila-pseudoobscura-female-reproductive-system-without-gonad	Drosophila-willistoni-female-thorax-without-digestive-system
dana-pseudogene-r1-06	Drosophila-ananassae-female-thorax-without-digestive-system	Drosophila-pseudoobscura-female-thorax-without-digestive-system	Drosophila-willistoni-male-abdomen-without-digestive-or-reproductive-system
dana-ncRNA-r1-06	Drosophila-ananassae-female-whole-body	Drosophila-pseudoobscura-female-whole-body	Drosophila-willistoni-female-whole-body
dana-miscRNA-r1-06	Drosophila-ananassae-male-abdomen-without-digestive-or-reproductive-system	Drosophila-pseudoobscura-male-abdomen-without-digestive-or-reproductive-system	Drosophila-willistoni-male-gonad
dana-tRNA-r1-06	Drosophila-ananassae-male-digestive-plus-excretory-system	Drosophila-pseudoobscura-male-digestive-plus-excretory-system	Drosophila-willistoni-male-digestive-plus-excretory-system
dmel-CDS-r6-41	Drosophila-ananassae-male-gonad	Drosophila-pseudoobscura-male-gonad	Drosophila-willistoni-male-head
dmel-chromosome-r6-41	Drosophila-ananassae-male-head	Drosophila-pseudoobscura-male-head	Drosophila-willistoni-male-reproductive-system-without-gonad
dmel-intergenic-r6-41	Drosophila-ananassae-male-reproductive-system-without-gonad	Drosophila-pseudoobscura-male-reproductive-system-without-gonad	Drosophila-willistoni-male-thorax-without-digestive-system
dmel-pseudogene-r6-41	Drosophila-ananassae-male-thorax-without-digestive-system	Drosophila-pseudoobscura-male-thorax-without-digestive-system	Drosophila-willistoni-male-whole-body
dmel-ncRNA-r6-41	Drosophila-ananassae-male-whole-body	Drosophila-pseudoobscura-male-whole-body	Drosophila-yakuba-female-abdomen-without-digestive-or-reproductive-system
dmel-miscRNA-r6-41	Drosophila-mojavensis-female-abdomen-without-digestive-or-reproductive-system	Drosophila-virilis-female-digestive-plus-excretory-system	Drosophila-yakuba-female-digestive-plus-excretory-system
dmel-miRNA-r6-41	Drosophila-melanogaster-male-whole-body	Drosophila-virilis-female-abdomen-without-digestive-or-reproductive-system	Drosophila-yakuba-female-gonad
dmel-intron-r6-41	Drosophila-mojavensis-female-gonad	Drosophila-virilis-female-head	Drosophila-yakuba-female-head
dmel-tRNA-r6-41	Drosophila-mojavensis-female-digestive-plus-excretory-system	Drosophila-virilis-female-gonad	Drosophila-yakuba-female-reproductive-system-without-gonad
dmel-transposon-r6-41	Drosophila-mojavensis-female-head	Drosophila-virilis-female-reproductive-system-without-gonad	Drosophila-yakuba-female-thorax-without-digestive-system
dsim-3prime-r2-02	Drosophila-mojavensis-female-reproductive-system-without-gonad	Drosophila-virilis-female-thorax-without-digestive-system	Drosophila-yakuba-female-whole-body
dsim-5prime-r2-02	Drosophila-mojavensis-female-thorax-without-digestive-system	Drosophila-virilis-male-abdomen-without-digestive-or-reproductive-system	Drosophila-yakuba-male-abdomen-without-digestive-or-reproductive-system
dsim-CDS-r2-02	Drosophila-mojavensis-female-whole-body	Drosophila-virilis-female-whole-body	Drosophila-yakuba-male-digestive-plus-excretory-system
dsim-pseudogene-r2-02	Drosophila-mojavensis-male-abdomen-without-digestive-or-reproductive-system	Drosophila-virilis-male-digestive-plus-excretory-system	Drosophila-yakuba-male-gonad
dsim-ncRNA-r2-02	Drosophila-mojavensis-male-digestive-plus-excretory-system	Drosophila-virilis-male-gonad	Drosophila-yakuba-male-head
dsim-miRNA-r2-02	Drosophila-mojavensis-male-gonad	Drosophila-virilis-male-reproductive-system-without-gonad	Drosophila-yakuba-male-reproductive-system-without-gonad
dsim-intron-r2-02	Drosophila-mojavensis-male-head	Drosophila-virilis-male-head	Drosophila-yakuba-male-thorax-without-digestive-system
dsim-rna-V3	Drosophila-mojavensis-male-reproductive-system-without-gonad	Drosophila-virilis-male-thorax-without-digestive-system	Drosophila-yakuba-male-whole-body
dyak-3prime-r1-05	Drosophila-mojavensis-male-thorax-without-digestive-system	Drosophila-willistoni-female-abdomen-without-digestive-or-reproductive-system	
dyak-5prime-r1-05	Drosophila-mojavensis-male-whole-body	Drosophila-virilis-male-whole-body	
dyak-CDS-r1-05	Drosophila-persimilis-female-abdomen-without-digestive-or-reproductive-system	Drosophila-persimilis-male-abdomen-without-digestive-or-reproductive-system	
dyak-ncRNA-r1-05	Drosophila-persimilis-female-digestive-plus-excretory-system	Drosophila-persimilis-male-digestive-plus-excretory-system	
dyak-miscRNA-r1-05	Drosophila-persimilis-female-gonad	Drosophila-persimilis-male-gonad	
dyak-miRNA-r1-05	Drosophila-persimilis-female-head	Drosophila-persimilis-male-head	
dyak-intron-r1-05	Drosophila-persimilis-female-reproductive-system-without-gonad	Drosophila-persimilis-male-reproductive-system-without-gonad	
dyak-tRNA-r1-05	Drosophila-persimilis-female-thorax-without-digestive-system	Drosophila-persimilis-male-thorax-without-digestive-system	
dyak-pseudogene-r1-05	Drosophila-persimilis-female-whole-body	Drosophila-persimilis-male-whole-body	
